# A Novel Biopsy Method Based on Bipolar Radiofrequency Biopsy Needles

**DOI:** 10.3389/fonc.2022.838667

**Published:** 2022-02-10

**Authors:** Huiyang Wang, Haiwei Bao, Lan Yue, Tian’an Jiang

**Affiliations:** Department of Ultrasound Medicine, The First Affiliated Hospital, Zhejiang University School of Medicine, Hangzhou, China

**Keywords:** biopsy, radiofrequency ablation, bleeding, anticoagulation, needle tract seeding

## Abstract

Modern oncology increasingly relies on pathological, molecular, and genomic assessments of biopsied tumor tissue. However, the concern for bleeding complication and malignant seeding severely hinders the application of the biopsy tumor. Here, we developed a 16 G biopsy needle to contain two electrodes insulated from each other and connect to an radiofrequency generator. For evaluating hemostatic efficacy, 50 rabbits were randomly divided into two groups: warfarinization and non-warfarinization group. Two liver biopsies and two splenic biopsies per animal were performed using a 16 G biopsy needle. Each group was further equally divided into five groups according to different hemostatic measures, including non-intervention, embolization using an absorbable gelatin sponge, and ablation by RF with three different needle temperatures (50°C, 70°C, and 90°C). Than, we used VX2 rabbit models (n = 25) and applied the five analogous biopsies to the tumor. The flush fluid from the biopsy needle underwent cytomorphological analysis. Our results that the groups using ablation by RF showed significantly less blood loss than the control group for liver and spleen in both groups (P < 0.001). After RF ablation, thermal coagulation of the tissue surrounding the needle tract was observed on both the macroscopic and histological level. Cytological smears showed that tumor cells were degenerated after RF at 70°C and 90°C. Our findings showed that bipolar RF biopsy needle is a promising tool for reducing hemorrhage after biopsy and avoiding implanting tumor cells in the tract.

## Introduction

Modern oncology increasingly relies on pathological, molecular, and genomic assessments of biopsied tumor tissue to guide treatment selection and evaluate therapeutic response or resistance (1). At present, there are several methods of biopsy available, such as surgical biopsy, core needle biopsy (CNB), and fine needle aspiration (FNA) biopsy (2). In general, more accurate and reliable the pathology results are obtained with more biopsy specimens of larger size (3–6). However, given the concern for bleeding complication, the size and number of biopsy specimens to be taken are limited. Bleeding tendencies (coagulation defects, decreased platelet counts, or long-term warfarin therapies) and organs susceptible to bleeding (spleen) are absolute contraindications for biopsy (7).

What is more, numerous case reports of malignant seeding resulting from needle biopsies have been reported for different tumors and increasing caution is being observed in highly aggressive malignancies (8–12). There is a growing consensus that the true incidence of tumor seeding along the needle may be underestimated as not all cases can be diagnosed, and many patients die before these metastases become clinically apparent (13).

Various approaches have been presented to reduce the risk of bleeding such as occluding the biopsy tract with various materials, as the needle is withdrawn (14–17). However, potential concerns remain regarding immunologic interactions with tissue-based agents and the technical complexity required to introduce the agent into the tract (18). Several radio-frequency (RF) ablation devices that can cauterize the tract for biopsy have been reported in recent years (19). However, these devices cannot display the needle temperature and RF power on the monitor in real-time. Laaseke et al. highlighted the importance of the incorporation of a real-time temperature feedback circuit to clinical thermal ablation systems (18).

In this study, we developed a biopsy needle featuring a fully integrated bipolar RF ablation electrode for the post-biopsy coagulation of the puncture tract. And the temperature sensor was incorporated close to the hot zone of the needle to monitor real-time needle temperature. The needle temperature in our system is controlled by thermocouple temperature sensor systems. The needle temperature and RF power are displayed on the monitor in real-time and can improve the operation experience and safety. The other purpose of this study was to verify its usefulness in reducing hemorrhage and dissemination of viable tumor cells in the biopsy tract.

## Materials and Methods

### Bipolar Radiofrequency Biopsy Needle System

**Figure 1:**
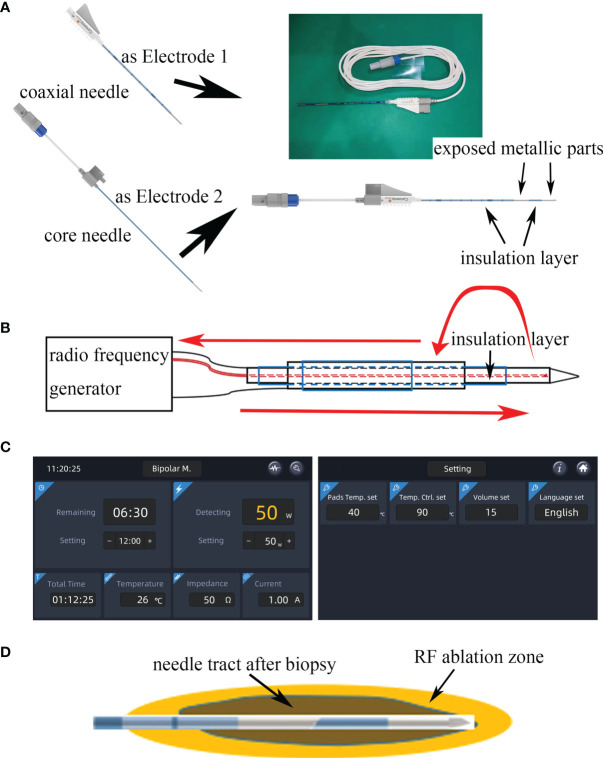
**(A)** The bipolar radiofrequency (RF) biopsy needle is composed of a coaxial needle (electrode 1) and core needle (electrode 2). The figure also shows the insulation and exposed metallic parts of the two needles. **(B)** RF signal from an RF generator s connected to the core needle. Current runs between the exposed metallic parts (red curved arrows) to reach the coaxial needle. **(C)** Real-time controlled user interface for needle temperature and power observations; **(D)** schematic of needle tract ablation.

The bipolar radiofrequency biopsy needle is composed of a coaxial needle (electrode 1) and core needle (electrode 2) as shown in **Figure 1A**. Both needles are coated with an insulating layer, but the tip is exposed as the conductive part. After taking the biopsy, the inner core needle is inserted into the outer tube needle to form a bipolar needle. Since the inner needle is slightly longer than the coaxial needle, there is an insulating layer between the exposed end of the inner needle and the coaxial needle when the inner needle is inserted. When the RF equipment (Zhejiang Curaway Medical Technology Inc., China) is running, the RF current will flow from electrode 2, cross the insulating layer, return to electrode 1, and form a closed circuit (**Figure 1B**). When the RF current flows through tissue, the charged molecules and ions in the tissue generate heat by friction and the temperature increases (**Figure 1D**). The needle ablation temperature is monitored by a thermocouple temperature sensor which is installed at the core needle tip. Based on the temperature, the RF generator controls the RF current output in real time through a proportional integral derivative (PID) algorithm, quickly reaches the set ablation control temperature and maintains the temperature. After the ablation hemostasis effect is achieved, a section of the RF generator is pulled according to the temperature of the new position, The RF current output is controlled in real time by the PID algorithm. After reaching the set ablation control temperature again and maintaining the temperature, ablation is continued and repeated until the ablation of the biopsy needle is completed. **Figure 1C** shows the bipolar mode display interface of the RF generator, and the main setting parameters are the power and temperature control settings.

### Animal Modeling

All male white New Zealand rabbits (1.8–2.2 kg) were purchased from the Experimental Animal Center of the Zhejiang Academy of Medicinal Sciences (Hangzhou, China). To evaluate the ability of reducing hemorrhage after biopsy and dissemination of viable tumor cells, we have designed two different animal models (warfarinization rabbit models and VX2 rabbit tumor models).

**Figure 2:**
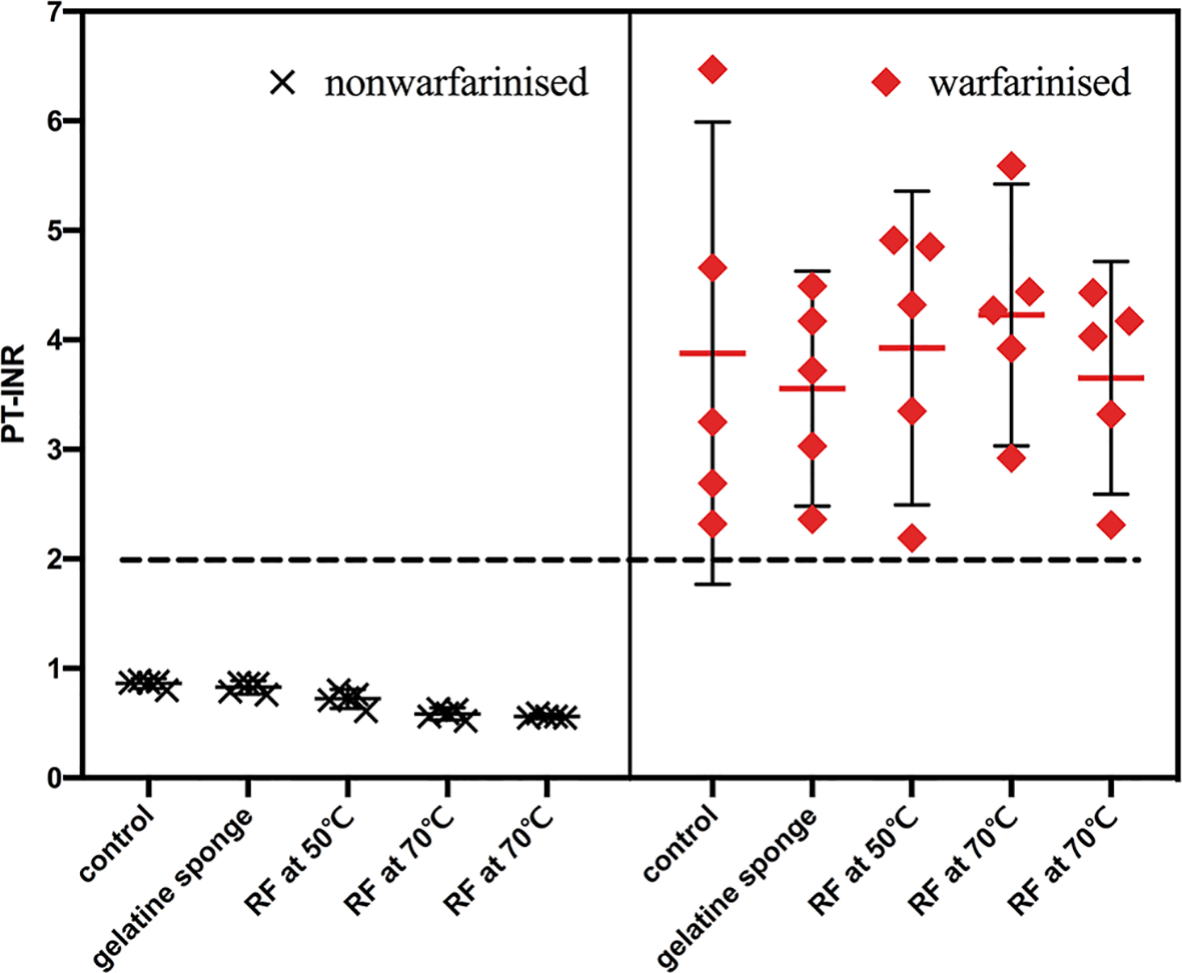
PT-INR of the 50 rabbit models were recorded before biopsy. PT-INR of the 25 rabbit models in the warfarinized group all reached 2, the other were within the normal range.

Warfarinization rabbit models (n = 25): warfarin sodium (Orion Pharma, Finland) was dissolved in 0.9% saline injection. The dose for rabbits was designed to be 0.25 mg/kg (20). The administration volume was 1 mL/kg, once a day for 3 days. In the clinic, the prothrombin time-international normalized ratio (PT-INR) level should be maintained above 2 for patients being treated with anticoagulation. Thus, PT-INR was dynamically monitored to adjust the dose of warfarin to a target of above 2 (21). The results showed the PT-INR of the 25 rabbit models all reached 2 before biopsy (**Figure 2**). The remaining coagulation function indexes were also dynamically monitored during administration, including prothrombin time (PT), and activated partial thromboplastin time (APTT).

VX2 rabbit tumor models (n = 25): One New Zealand white rabbit bearing VX2 tumor in the erector spinae muscle was provided by Hangzhou Shuguang Technology Co., Ltd. (Hangzhou, China). The VX2 tumor tissue obtained from the tumor-bearing rabbit was resected, cut with scissors, and minced into 1×1×1 mm3 fragments. After anesthesia using intramuscular injection of xylazine hydrochloride (10 mg/kg) (Jilin Animal Husbandry Animal Health Products Co. Ltd., China), rabbits were placed in a supine position. The right hind leg was shaved and disinfected with 7.5% povidone-iodine solution. The skin and muscle layer of the lateral thigh were cut in turn. The muscle layer was implanted with a VX2 tumor fragment. The incision was then sutured and disinfected.

### Biopsy Protocol

In the first part of the study, 50 rabbits were randomly divided into two groups: warfarinization and non-warfarinization group. Two liver biopsies and two splenic biopsies per animal were performed using a 16 G biopsy needle, leading to a total of 200 biopsies (100 for liver and 100 for spleen). Each group was further equally divided into five groups according to different hemostatic measures, including non-intervention, embolization using an absorbable gelatin sponge, and ablation by RF with three different needle temperatures (50°C, 70°C, and 90°C). During the biopsy procedures of both study and control groups, a 15 G coaxial introducer needle containing electrode 1 (ie, a hollow metal tube) and electrode 2 (ie, an inner stylus) inserted into the rabbit’s liver or spleen at an approximately 3 cm depth. After the coaxial needle was placed, electrode 2 was withdrawn, leaving only the electrode 1 in place. Next, an 16 g cutting needle in an automatic biopsy gun (Zhejiang Curaway Medical Technology Inc., China) was was inserted through the coaxial introducer needle to collect the biopsy samples. For the control group, after the biopsy sample was collected, the core biopsy needle was reinserted through the coaxial introducer needle and removed together with the coaxial introducer needle to mimic a standard biopsy procedure, whereas for the second group, after the biopsy sample was collected, gelatin sponge granules were pushed in the core of the needle to stop bleeding. For the third, fourth, and fifth groups, electrode 2 as the RF ablation needle was inserted through the coaxial introducer needle to perform biopsy tract ablation. During tract ablation, the RF power was turned on and the needle was held stationary until it reached the target temperature as displayed on the monitor. The needle was then slowly retracted until the needle was completely removed from the liver or spleen. The duration of the procedure was recorded by the operator performing the study using a digital stopwatch.

The amount of blood at each biopsy site was collected using dry cotton ball until the bleeding completely stopped. The amount of blood loss was determined as follows: blood weight (g)  =  wet cotton ball weight − dry cotton ball weight. Weights were measured with an electronic balance with 1 mg resolution (YH-A 6002, Yingheng Electric Appliance Co. Ltd., China).

Rabbits were euthanized by IV overdose of xylazine hydrochloride (50 mg/kg). The livers and spleens were then harvested and sectioned along the axial plane of the biopsy tract. The ablation area widths in the liver or spleen were measured and recorded. All tissues were fixed with 10% neutral formalin for 1 day and washed with PBS. The specimens were then embedded in paraffin and sliced into 4-µm sections for hematoxylin and eosin (H&E) staining.

In the second part of the study, 25 VX2 tumor-bearing rabbits (approximately 10 mm tumor diameter) were randomly divided into five groups: the control group (n = 5), absorbable gelatin sponge packing group (n = 5), and the RF ablation groups with different needle temperatures of 50°C (n = 5), 70°C (n = 5), and 90°C (n = 5). Five tumor biopsies were done per rabbit, leading to a total number of 125 biopsies. All biopsies were performed under ultrasound guidance (Z-One, Mindray Bio-Medical Electronics Co., Ltd., China). Tumor biopsies and hemostatic measures of different groups were performed in the same manner as in the hemostatic efficacy evaluation. Afterward, the biopsy needle flush fluid was collected by repeatedly flushing the needle with 1 mL of neutral buffered formalin containing 4% formaldehyde. Subsequently, the flush fluid were placed in a 4°C refrigerator for 24 h. The samples then underwent centrifugation (1000 rpm) for 5 min at 4°C (TDZ5-WS, Changsha Xiangyi Centrifuge Instrument Co., Ltd., China). The supernatant was discarded, and the cells were re-suspended in 0.5 mL of neutral buffered formalin. Then, smears were manually prepared by spreading a drop of suspension on a glass slide and stained using H&E staining for cytological analysis.

### Statistical Analysis

All results are reported as the mean ± standard deviation. Data were analyzed with SPSS version 26 or GraphPad Prism software version 8.3.0. Tests for significant differences in blood losses in the control and post-biopsy bleeding control groups were performed using repeated measurements analysis of variance. *Post hoc* comparisons were done using the Tukey–Kramer test. Differences in bleeding amounts in warfarinized and non-warfarinized animals were assessed by the Student’s *t*-test. A *P* value of < 0.05 was considered statistically significant.

## Results

### Comparison of Differences in Blood Loss

**Figure 3:**
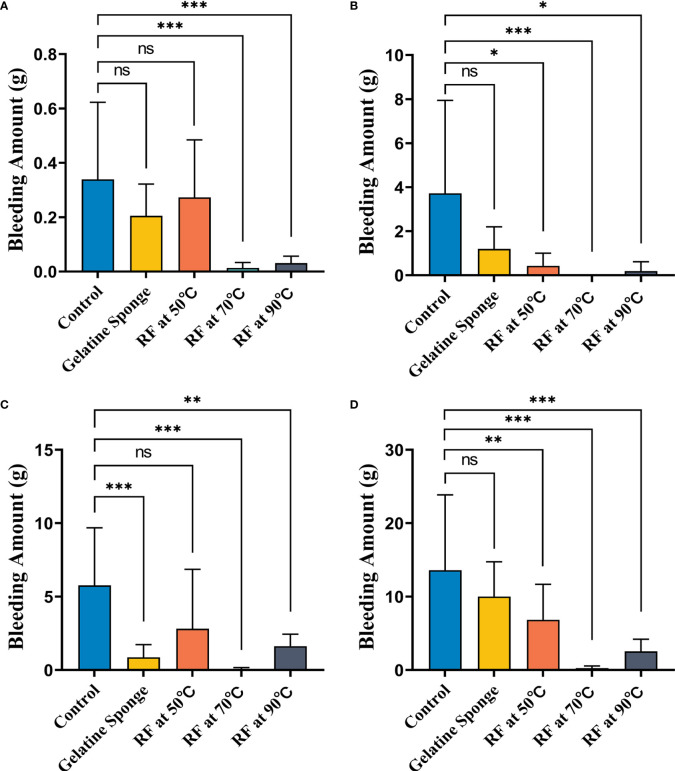
Comparison of differences in blood loss (g) between five different hemostatic measures after biopsy **(A)** in the non-warfarinized group for livers, **(B)** warfarinized group for livers, **(C)** non-warfarinized group for spleens, and **(D)** warfarinized group for spleens. ns, P > 0.05 vs control,*P < 0.05 vs control, **P < 0.01 vs control, ***P < 0.001 vs control.

The results of the Tukey–Kramer test for multiple comparisons of post-biopsy bleeding amounts are illustrated in **Tables 1**, **2** and **Figure 3**.

**Table 1:** Bleeding amounts for the liver.

Group and procedure	Mean bleeding difference (g)	*P*
**Non-warfarinized**		
Control vs. gelatin sponge	0.134 (−0.791 – 0.347)	0.395
Control vs. RF at 50˚C	0.066 (−0.147 – 0.279)	0.904
Control vs. RF at 70˚C	0.326 (0.113 – 0.538)	0.001
Control vs. RF at 90˚C	0.308 (0.095 – 0.521)	0.001
Gelatin sponge vs. RF at 50˚C	0.068 (−0.281 – 0.145)	0.892
Gelatin sponge vs. RF at 70˚C	0.192 (−0.021 – 0.405)	0.095
Gelatin sponge vs. RF at 90˚C	0.174 (−0.039 – 0.387)	0.155
RF at 50˚C vs. RF at 70˚C	0.260 (0.047 – 0.473)	0.010
RF at 50˚C vs. RF at 90˚C	0.242 (0.029 – 0.455)	0.018
RF at 70˚C vs. RF at 90˚C	−0.018 (−0.230 – 0.195)	0.999
**Warfarinized**		
Control vs. gelatin sponge	2.520 (−0.210 – 5.249)	0.083
Control vs. RF at 50˚C	3.285 (0.555 – 6.014)	0.011
Control vs. RF at 70˚C	3.719 (0.989 – 6.448)	0.003
Control vs. RF at 90˚C	3.027 (−0.297 – 5.756)	0.023
Gelatin sponge vs. RF at 50˚C	0.765 (−1.964 – 3.494)	0.930
Gelatin sponge vs. RF at 70˚C	1.199 (−1.530 – 3.929)	0.723
Gelatin sponge vs. RF at 90˚C	0.507 (−2.222 – 3.236)	0.984
RF at 50˚C vs. RF at 70˚C	0.434 (−2.295 – 3.163)	0.991
RF at 50˚C vs. RF at 90˚C	−0.258 (−2.988 – 2.471)	0.999
RF at 70˚C vs. RF at 90˚C	−0.692 (−3.422 – 2.037)	0.951

Dates for the bleeding amount are mean (95% confidence interval). RF, radiofrequency cauterization.

**Table 2:** Bleeding amounts for the spleen.

Group and procedure	Mean bleeding difference (g)	*P*
**Non-warfarinized**		
Control vs. gelatin sponge	4.903 (1.630 – 8.176)	0.001
Control vs. RF at 50˚C	2.954 (−0.319 – 6.227)	0.095
Control vs. RF at 70˚C	5.765 (2.492 – 9.038)	0.000
Control vs. RF at 90˚C	4.137 (0.864 – 7.409)	0.007
Gelatin sponge vs. RF at 50˚C	−1.950 (−5.223 – 1.323)	0.449
Gelatin sponge vs. RF at 70˚C	0.861 (−2.412 – 4.134)	0.944
Gelatin sponge vs. RF at 90˚C	−0.767 (−4.040 – 2.506)	0.963
RF at 50˚C vs. RF at 70˚C	2.811 (−0.462 – 6.083)	0.123
RF at 50˚C vs. RF at 90˚C	1.183 (−2.090 – 4.456)	0.842
RF at 70˚C vs. RF at 90˚C	−1.628 (−4.901 – 1.645)	0.622
**Warfarinized**		
Control vs. gelatin sponge	5.598 (−0.864 – 12.060)	0.118
Control vs. RF at 50˚C	8.754 (2.292 – 15.216)	0.003
Control vs. RF at 70˚C	15.333 (8.871 – 21.795)	0.000
Control vs. RF at 90˚C	13.056 (6.594 – 19.518)	0.000
Gelatin sponge vs. RF at 50˚C	3.156 (−3.306 – 9.618)	0.638
Gelatin sponge vs. RF at 70˚C	9.735 (3.273 – 16.197)	0.001
Gelatin sponge vs. RF at 90˚C	7.458 (0.996 – 13.920)	0.016
RF at 50˚C vs. RF at 70˚C	6.579 (0.117 – 13.041)	0.044
RF at 50˚C vs. RF at 90˚C	4.302 (−2.160 – 10.764)	0.336
RF at 70˚C vs. RF at 90˚C	−2.276 (−8.739 – 4.185)	0.853

Dates for the bleeding amount are mean (95% confidence interval). RF, radiofrequency cauterization.

In the non-warfarinized group’s liver results, needle temperatures at 70°C and 90°C differed significantly from the control group (P < 0.05). The gelatin sponge group and 50°C needle temperature group did not differ from the control group (P > 0.05). In the warfarinized group’s liver results, all radiofrequency cauterization statistically reduced bleeding compared with the control (P < 0.05). However, the gelatin sponge group also did not differ from the control group (P > 0.05).

In the non-warfarinized group’s spleen results, while RF at 50°C did not show any differences compared with the controls (P > 0.05), biopsy sites after using the gelatin sponge and RF at 70°C and 90°C had significantly less blood loss than did control biopsy sites (P < 0.05). In the warfarinized group’s spleen results, all rabbits had abundant bleeding in both surgeries. From a statistical perspective, RF ablation is significantly superior in reduction bleeding. All needle temperature groups at 50°C, 70°C, and 90°C differed significantly from the control group (P < 0.05).

### Macroscopic Assessments

**Figure 4:**
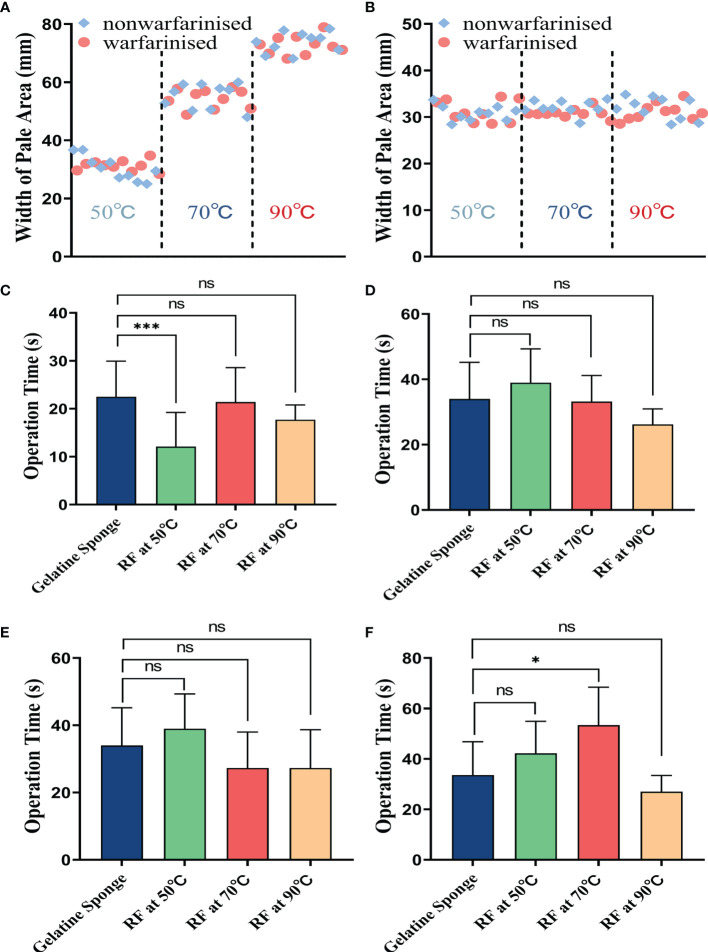
The tract ablation area width at different needle temperatures in the liver **(A)** or spleen **(B)**. Comparison of difference in operation time(s) between four different hemostatic measures after biopsy, including: **(C)** in the non-warfarinized group for livers, **(D)** warfarinized group for livers, **(E)** non-warfarinized group for spleens, and **(F)** warfarinized group for spleens. Due to not having a hemostatic measure, the control group is not included in the comparison. ns P > 0.05 vs control, *P < 0.05 vs control, **P < 0.01 vs control, ***P < 0.001 vs control.

The coagulated puncture tracts after RF at different needle temperatures were evaluated by a combination of macroscopic and histological examination. The macroscopic evaluation showed a clear pale area surrounding the needle tract. At different temperatures (50°C, 70°C, and 90°C), the mean (± SD) width of the liver pale area in the non-warfarinized group was 30.41 ± 3.97 mm, 55.22 ± 4.19 mm, and 73.76 ± 3.38 mm, respectively. In the warfarinized group, the widths were 31.30 ± 1.77 mm, 54.40 ± 0.95 mm, and 72.68 ± 3.12 mm, respectively. For the spleen in the non-warfarinized group, the widths were 30.85 ± 1.53 mm, 32.10 ± 1.43 mm, and 31.91 ± 2.26 mm, respectively, at different temperatures. In the warfarinized group, the widths were 31.26 ± 2.23 mm, 30.83 ± 0.95 mm, and 31.13 ± 1.73 mm, respectively. **Table 3** shows that no significant differences were found between the non-warfarinized and warfarinized groups at the same needle temperature for liver or spleen (P > 0.05). Moreover, we found a positive significant correlation between the width of the pale area and needle temperature in the liver but did not see any correlation between the two in the spleen (**Figures 4A, B**).

**Table 3:** Results for pale area width.

Group and procedure	Mean Width Difference (mm)	*P*
**RF at 50˚C**		
Non-warfarinized vs. warfarinized in liver	−0.890 (−4.508 – 2.728)	0.591
Non-warfarinized vs. warfarinized in spleen	−0.418 (−1.532 – 0.696)	0.418
**RF at 70˚C**		
Non-warfarinized vs. warfarinized in liver	0.819 (−2.347 – 3.985)	0.573
Non-warfarinized vs. warfarinized in spleen	1.273 (−0.097 – 2.643)	0.065
**RF at 90˚C**		
Non-warfarinized vs. warfarinized in liver	1.072 (−2.763 – 4.907)	0.543
Non-warfarinized vs. warfarinized in spleen	0.786 (−1.625 – 3.197)	0.480
**Non-warfarinized in liver**		
RF at 50˚C vs. RF at 70˚C	−24.807 (−29.29 – 20.315)	0.000
RF at 50˚C vs. RF at 90˚C	−43.341 (−48.360 – 38.321)	0.000
RF at 70˚C vs. RF at 90˚C	−18.534 (−22.899 – 14.169)	0.000
**Warfarinized in liver**		
RF at 50˚C vs. RF at 70˚C	−23.098 (−25.647 – 20.549)	0.000
RF at 50˚C vs. RF at 90˚C	−41.379 (−44.179 – 38.579)	0.000
RF at 70˚C vs. RF at 90˚C	−18.281 (−21.308 – 15.253)	0.000
**Non-warfarinized in spleen**		
RF at 50˚C vs. RF at 70˚C	−1.258 (−2.748 – 0.232)	0.088
RF at 50˚C vs. RF at 90˚C	−1.068 (−3.357 – 1.221)	0.319
RF at 70˚C vs. RF at 90˚C	0.190 (−1.526 – 1.906)	0.808
**Warfarinized in spleen**		
RF at 50˚C vs. RF at 70˚C	0.433 (−1.274 – 2.140)	0.580
RF at 50˚C vs. RF at 90˚C	0.136 (−2.016–2.288)	0.889
RF at 70˚C vs. RF at 90˚C	−0.297 (−1.480 – 0.886)	0.584

Dates for the width difference are mean (95% confidence interval). RF, radiofrequency cauterization.

### Comparison of Differences in Operation Time

**Figures 4C–F** shows the results of the comparison of operation times for different hemostatic measures. In the livers of the non-warfarin group, there were no significant differences between the 70°C and 90°C RF group and the gelatin sponge packing group (P > 0.05). In the livers of the warfarin group and the spleens of the non-warfarin group, there were no significant differences between all the RF groups and gelatin sponge packing group (P > 0.05). In the warfarinized spleen group, there was no significant difference between the 50°C and 90°C RF groups compared to the gelatin sponge packing group (P > 0.05). The operation time of the RF group at needle temperature of 70°C was slightly longer than that of the gelatin sponge packing group (P < 0.05).

### Histological Assessments

**Figure 5:**
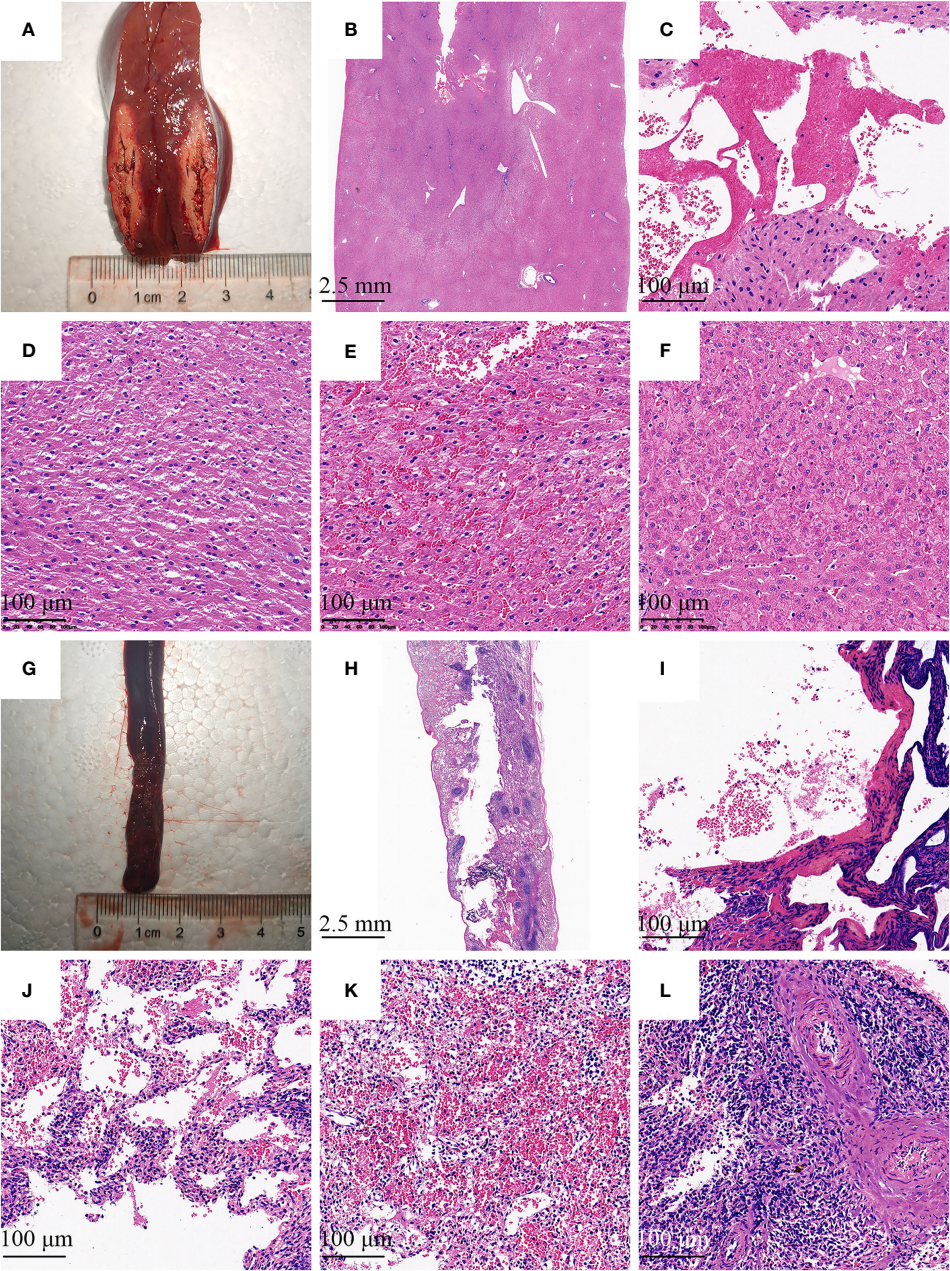
Histologic image of the needle tract ablation specimen. Photographs show sections along the needle tract of liver **(A)** and spleen **(G)** after needle removal. The needle tract center to pale area represents the coagulation of the tissue. **(B, H)** There were four distinguished zones in the liver and spleen, including a central cavity, weak eosin staining area, deep red peripheral zone, and normal tissue region. **(C, I)** Tissue had been lost and filled with fibrins and red blood cells induced by RF. **(D, J)** Decrease in intensity of eosin staining was observed with detachment of cells. **(E, K)** The hemorrhagic rim in the surrounding of ablation area. **(F, L)** Normal liver or spleen tissue region that is at further distance from the ablation area. **(B, H)** H&E stain with 4 × magnification; **(C–F)**, and **(I–L)** H&E stain with 100× magnification.

Histological alterations were evaluated with H&E staining. **Figure 5** showed there were four distinguished zones in the liver and spleen. Zone 1 was a central cavity, where tissue had been lost, and filled with fibrins and red blood cells induced by RF. Zone 2 showed typical coagulation necrosis of the ablated lesions, including cell shrinkage, weak eosin staining, and increased extracellular space. A thin peripheral zone (zone 3) contained a hemorrhagic rim with focal sinusoidal hemorrhage. Zone 4 was the normal tissue region located away from the ablated region.

### Cytological Analysis

**Figure 6:**
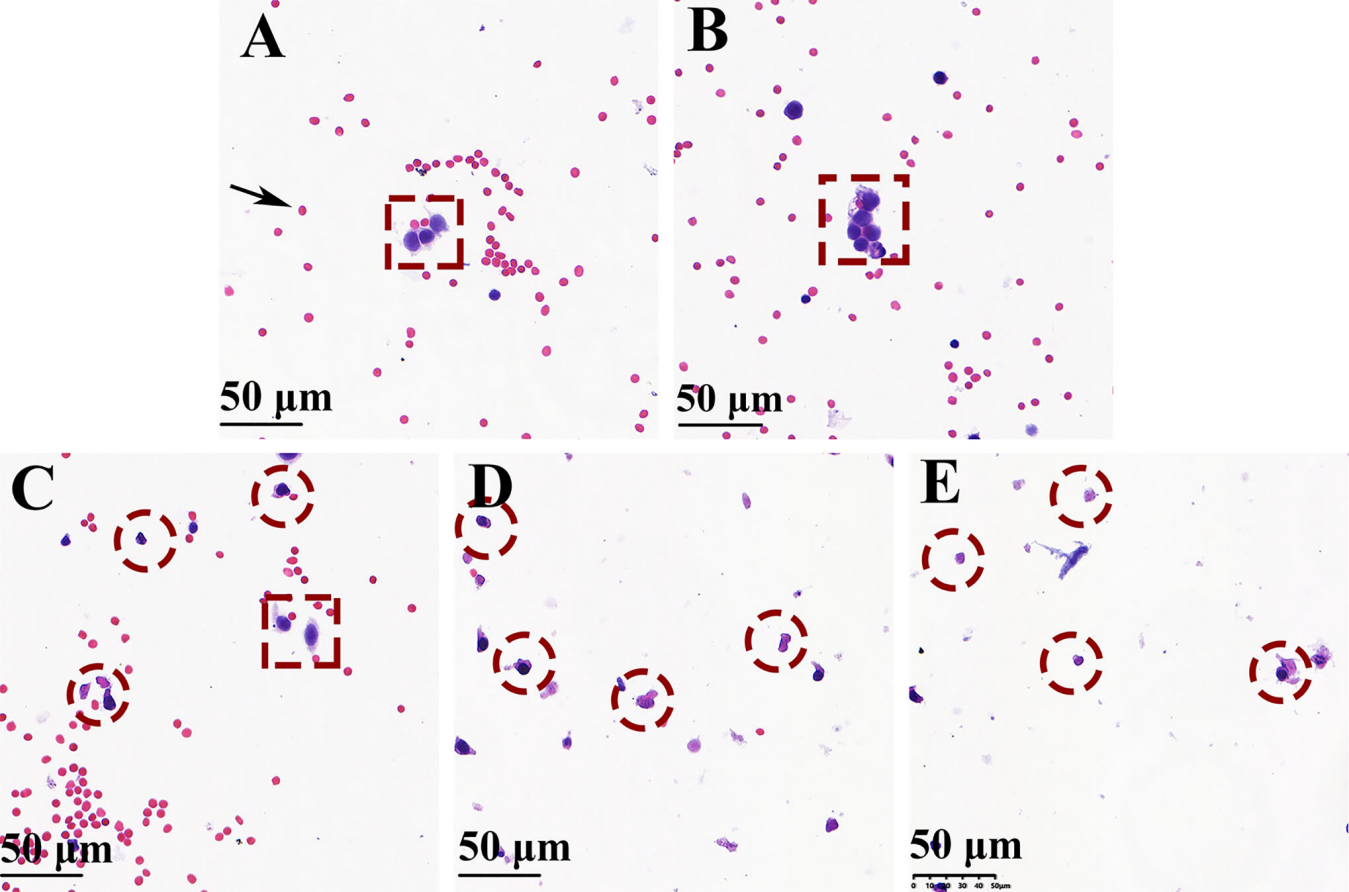
H&E-stained cell smear of biopsy needle flush fluid showing blood cells (black arrow), normal cancer cells (red dotted box), and denatured cancer cells (red-dotted circle) (magnification × 200). **(A, B)** Well-preserved cancer cells were seen surrounded by blood cells without using the bipolar radiofrequency biopsy needle (groups 1 and 2). **(D, E)** Only a minor number of blood cells and denatured cancer cells were observed when RF biopsy needle temperature reach above 70°C (groups 4 and 5). **(C)** Ablation performed at 50°C of needle temperature, the cell smear shows the presence of both normal and degenerationcancer cells.

**Figure 6A, B** show the cell smears from groups 1 and 2, respectively. The cancer cells are tagged with red dotted boxes, which adhered with each other and showed hyperchromatic pleomorphic nuclei. The cytological analysis of the cells after RF at 70°C and 90°C showed a significantly reduced number of erythrocytes, and all cancer cells indicated by red-dotted circles were judged as cytomorphologically degenerated (**Figure 6D, E**). However, when the needle temperature was 50°C, cancer cell partial inactivation was seen (**Figure 6C**).

## Discussion

Post-biopsy bleeding and needle tract seeding during biopsies are recognised complications of these procedures and a cause of concern in certain aggressive tumour forms. Ryosuke et al. reported that FNB-related needle tract seeding was detected in 2.7% of the patients with solid pancreatic masses (10). Considering that serial sections of whole resected specimens are microscopically examined at 5-mm intervals, needle tract seeding can be expected to occur more frequently. What is more, based on the report by Levy et al., it is possible that cancer cells translocate from the puncture route to the peritoneal cavity, leading to peritoneal dissemination (22). Thus, an aggressive approach in the diminishes the risk of tumour cell seeding as we have taken herein seems warranted. Although tract ablation is considered to be an effective mean for reducing hemorrhage after biopsy and dissemination of viable tumor cells in the tract, it is rarely performed in most clinical settings (23). This lack of application of radiofrequency energy to occlude the biopsy tract is multifactorial, with key limitations including time cost, lack of expertise, and lack of certain devices.

In our study, we developed a new biopsy needle to fully integrate the RF coagulation system into the biopsy device. Owing to its ease and speed of use and high rate of transmural completeness, the RF bipolar ablation device was selected for application in a new style of biopsy needle. Theoretically, thermal damage occurs when the local temperature is increased to 50°C in the liver, pancreas, kidney, and spleen (24). Temperatures above 60°C will cause instantaneous coagulation necrosis to stop bleeding, and temperatures above 105°C will cause tissue vaporization. Thus, the RF power must be corrected according to the real-time needle temperature. In our device, the needle temperature and RF power are displayed on the monitor in real-time and can thus improve the operation experience and safety. When the needle temperature rises above 70°C, a beeping alarm will remind the user to decrease the RF power.

It is well known that severe coagulation dysfunction is a taboo for biopsy. In the first part, we also artificially created a coagulopathic state in 25 rabbits by the administration of warfarin. In both the non-warfarinized and warfarinized groups, when the needle temperature rose above 70°C, radiofrequency cauterization significantly reduced bleeding at the biopsy site compared with the controls. So these patients with severe coagulopathy would greatly benefit from this technique and device that could reduce hemorrhage. In addition, this technology and device can also be used to improve splenic biopsy, which is a procedure with potential complications, such as hemorrhage.

In the second part of the study, using the VX-2 tumor as a model in this feasibility study proved suitable. After needle radiofrequency ablation, as no well-preserved tumor cells were present in the washes of the biopsy needle, it was reasonable to assume that they were denatured in the needle track. This technology should be applicable for the biopsy of a variety of cancer types, particularly in highly aggressive, metastatic phenotype, and poor prognosis.

The results of our study in the liver and spleen, even those in the anticoagulated state, are encouraging. This device has considerable utility in the biopsy. However, this application needs further large-scale experimental and clinical studies.

## Conclusion

In summary, we developed a new type of biopsy needle featuring a fully integrated bipolar RF ablation electrode with real-time thermal feedback. In this study, when the needle temperature rose above 70°C, RF cauterization offers effective bleeding control and shortens the procedure time. What is more, the presented technology could denature cancer cells in the needle track. Thus, bipolar RF biopsy needle is a promising tool for reducing hemorrhage after biopsy and dissemination of viable tumor cells in the tract.

## Data Availability Statement

The original contributions presented in the study are included in the article. Further inquiries can be directed to the corresponding author.

## Ethics Statement

The animal study was approved by the Institutional Animal Care and Use Committee, Zhejiang Center of Laboratory Animals (approval No. ZJCLA-IACUC-20030022).

## Author Contributions

T’AJ designed the experiments. HW and HB conducted the experiments. HW, HB, and LY analyzed the data. All authors contributed to the article and approved the submitted version.

## Funding

This study was supported by Development Project of National Major Scientific Research Instrument (82027803), National Natural Science Foundation of China (81971623), and Key Project of Natural Science Foundation of Zhejiang Province (LZ20H180001).

## Conflict of Interest

The authors declare that the research was conducted in the absence of any commercial or financial relationships that could be construed as a potential conflict of interest.

## Publisher’s Note

All claims expressed in this article are solely those of the authors and do not necessarily represent those of their affiliated organizations, or those of the publisher, the editors and the reviewers. Any product that may be evaluated in this article, or claim that may be made by its manufacturer, is not guaranteed or endorsed by the publisher.
